# The complete chloroplast genome sequence of *Ficus formosana* Maxim (Moraceae) from Guangzhou, China

**DOI:** 10.1080/23802359.2021.1934170

**Published:** 2021-06-07

**Authors:** Shiqiang Xu, Song Guo, Dongdong Fan, Jihua Wang

**Affiliations:** a Guangdong Provincial Key Laboratory of Crops Genetics and Improvement, Crops Research Institute, Guangdong Academy of Agricultural Sciences, Guangzhou, China; b College of Food and Biochemical Engineering, Guangxi Science and Technology Normal University, Laibin, China; c Key Laboratory for Research and Development of Characteristic Yao Medicine Resources, Guangxi Science and Technology Normal University, Laibin, China; d Shifang Municipal Bureau of Agriculture and Rural Affairs, Shifang, China

**Keywords:** *Ficus formosana*, chloroplast genome, medical plant, phylogenetic analysis

## Abstract

*Ficus formosana* Maxim is an economically valuable plant that is traditionally used in Chinese medicine. Here, we assembled the complete chloroplast (cp) genome of *F. formosana* using Illumina high-throughput sequencing technology. The cp genome size is 160,606 bp, with 35.90% GC content, including a large single copy region (LSC) of 88,668 bp, a small single copy region (SSC) of 20,140 bp and a pair of inverted repeat regions (IRs) of 25,899 bp. It encodes 86 protein-coding, 37 tRNA and 8 rRNA genes. Phylogenetic analysis fully resolved *F. formosana* on a branch with *Ficus erecta* within the genus *Ficus*. The complete cp genome sequence of *F. formosana* will provide valuable information for species identification and phylogenetic evolution.

*Ficus formosana* Maxim is a small evergreen tree, classified in the genus *Ficus* in the Moraceae. It is mainly distributed in South China, such as Taiwan, Fujian and Guangdong province (Huang et al. 2013). . Its dried roots are an excellent traditional Chinese herbal medicine, which have various activities, such as promoting blood circulation, relieving cough, expelling wind and dampness, anti-inflammatory, and antitumor (Sheu et al. 2005; Huang et al. 2013). In addition, it is a supplement in soups and gives off a special aroma that commonly used in medicinal diet (Wu et al., 2011). *F. formosana* also has great ornamental value and is often used to make bonsai. In addition, *F. formosana* has become useful in horticulature for preventing soil erosion in mountainous areas of Southern China due to its well developed roots system (Li et al., 2011).

Although studies of *F. formosana* using genetic markers have been published (Li et al., 2012), the cp genome of *F. formosana* has not been assembled. To investigate its chloroplast genome structure and content, and evolutionary relationship to other Moraceae, we assembled its cp genome and inferred its phylogenetic history.

Fresh leaves of *F. formosana* were collected from Guangdong Academy of Agricultural Sciences (Guangzhou, China；N23.1459, E113.3498), and were used for genomic DNA extraction by the CTAB method (Doyle and Doyle, 1990). The herbarium voucher specimen is deposited at the Medicinal Plant Germplasm Resource Nursery (Jihua Wang, wangjihua@gdaas.cn). The DNA is stored at the Key Laboratory for Crops Genetic Improvement of Guangdong in Guangdong Academy of Agricultural Sciences (specimen code Twr2020). The genomic sequencing library was constructed and seuquenced on the Illumina Novaseq platform (Illumina, San Diego, CA). About 6.0 Gb raw sequence reads were obtained and were filtered by Trimmomatic v.0.33 (Bolger et al. 2014). The cp genome was assembled and annotated by GetOrganelle v1.6.2e and Geseq, respectively (Tillich et al. 2017; Jin et al. 2020).

The total length of *F. formosana* cp genome is 160,606 bp, with 35.90% GC content, comprising a pair of IRs 25,899 bp, separating the LSC region of 88,668 bp and SSC region of 20,140 bp (GenBank assession number MW648426). It contains 84 protein-coding, 37 tRNA and 8 rRNA genes. In addition, there are 18 genes containing introns, including 13 in the LSC region, one in the SSC region and four in the IRs region. These values were similar to the cp genomes of other *Ficus* species (Chen et al. 2020; Wang and Cui 2020). 

To determine the phylogenetic position of *F. formosana*, fourteen species within *Ficus* with three outgroup taxa (*Broussonetia kaempferi*, *Morous mongolica* and *Trophis caucana*) were used for phylogenetic analysis. The phylogenetic tree was constructed using maximum-likelihood method by RaxML v8.2.12 software under the automatically selected model for 1,000 bootstraps (Stamatakis 2014). As shown in Figure 1, *F**. formosana* was resolved within *Ficus* on a branch with *F. erecta*. This result was consistent with the previous report (Li et al., 2012). In conclusion, the characterized cp genome sequence of *F. formosana* will provide a useful genetic resource for future phylogenetic relationship and identification of *F. formosana*.

**Figure 1.**
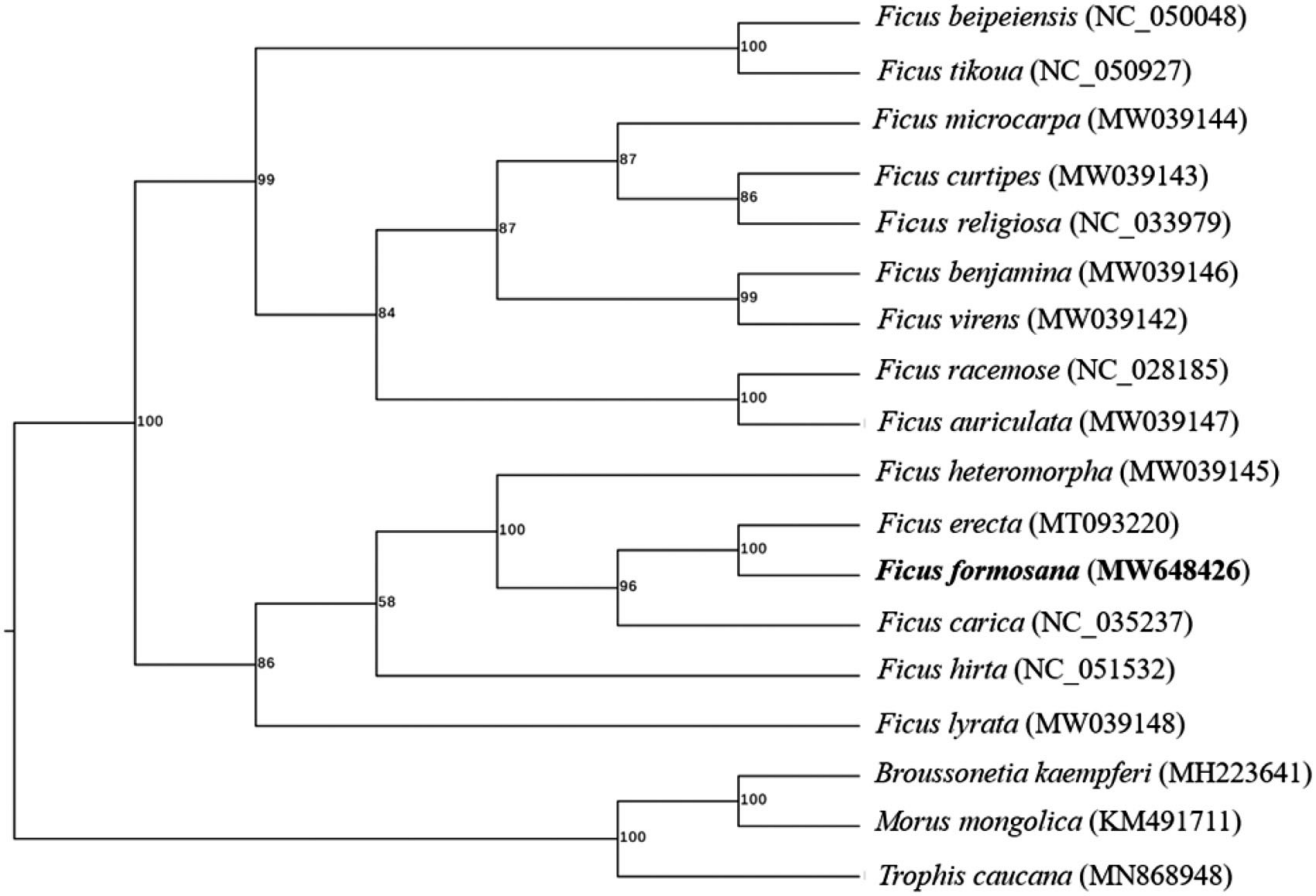
The phylogenetic tree of *F. formosana* with other species based on the complete chloroplast sequences. Numbers above each node are bootstrap values based on 1,000 replicates.

## Data Availability

The genome sequence data that support the findings of this study are openly available in GenBank of NCBI at [https://www.ncbi.nlm.nih.gov] (https://www.ncbi.nlm.nih.gov/) under the accession no. MW648426. The associated BioProject, SRA, and BioSample numbers are PRJNA714733, SRP310784, and SAMN18316700, respectively. The genome sequence data can be accessed via accession number MW648426 in GenBank of NCBI at https://www.ncbi.nlm.nih.gov. The associated SRA numbers is PRJNA714733.
